# Supplementary material for: Nicotinic Acid Catabolism Modulates Bacterial Mycophagy in Burkholderia gladioli Strain NGJ1
Source: Microbiol Spectr. 2023 Apr 4;11(3):e04457-22. doi: 10.1128/spectrum.04457-22 (PMC10269826; doi:10.1128/spectrum.04457-22)
Supplement: Supplemental file 1 — Supplemental material. Download spectrum.04457-22-s0001.pdf, PDF file, 0.7 MB [file spectrum.04457-22-s0001.pdf]

Table S1: *B. gladioli* strain NGJ1 harbors NA catabolic *nic* genes.

**Locus ID #**		**Gene product**	**% Identity at amino acid level**		
**Nucleotide ID**	**Protein ID**		***B. gladioli* BSR3**	***B. bronchiseptica* RB50**	***P. putida* KT2440**
ACI79_RS00350	WP_047835330.1	(2Fe-2S)-binding protein (*nicA*)	98.80	62.75	69.59
ACI79_RS00355	WP_047835331.1	cytochrome C (*nicB1*)	97.97	49.64	43.32
ACI79_RS00320	WP_047835539.1	aldehyde dehydrogenase (*nicB2*)	98.07	61.53	-
ACI79_RS00345	WP_013697295.1	6-hydroxynicotinate 3-monooxygenase (*nicC*)	100.00	69.62	64.80
ACI79_RS00340	WP_013697294.1	Asp/Glu racemase (*nicE*)	100.00	82.00	70.00
ACI79_RS00335	WP_047835329.1	alpha/beta hydrolase (*nicD*)	98.21	69.49	58.61
ACI79_RS00330	WP_047835328.1	2,5-dihydroxypyridine 5,6-dioxygenase (*nicX*)	99.71	79.01	55.56
ACI79_RS00325	WP_047835540.1	N-carbamoylsarcosine amidase (*nicF*)	99.07	70.79	40.84
ACI79_RS00315	WP_013697289.1	MarR family transcriptional regulator (*nicR*)	100.00	59.21	53.47

Locus and protein ids are as per Burkholderia Genome Database (https://www.burkholderia.com/)

Table S2. Bacterial strains and plasmids used in this study

**Strains or plasmid**	**Relevant characteristics**	**Source/reference**
**Fungal strains**		
Rhizoctonia solani (BRS1)	AG1-IA strain; an Indian isolate	Lab collection
**Bacterial strains**		
E. coli **strains**		
DH5α	F', endA1 hsdR17 (rk- mk+) supE44 thi-1 recA1 gyrA TelA1 cp8OdlacZAM15 A (lacZYA-argF) U169	Lab collection
TP003	S17-1 carrying *Tn5gusA11* in a suicide plasmid	Lab collection
B. gladioli **strains**		
NGJ1	Rif^r derivative	Lab collection
Δ*nicC*	pK18mob::*nicC*; Km^r derivative of NGJ1 (Δ*rpoN1* mutant strain)	Current study
Δ*nicX*	pK18mob::*nicX*; Km^r derivative of NGJ1 (Δ*rpoN2* mutant strain)	Current study
Δ*nicR*	pK18mob::*nicR*; Km^r derivative of NGJ1 (Δ*rpoN2* mutant strain)	Current study
**Plasmids**		
pBluescript KS(+)	pUC19 derivative; (2.958 kp); Amp^r	Lab collection
pK18mob	pUC18 derivative; (3.793 kb); Mob+ Tra– Km^r	Lab collection

Table S3. Primers used in this study

Gene / gene product	Primer IDs	Primer sequence
**Mutational analysis**		
nicC partial fragment cloning in pK18mob	nicC F	GATTTCCACGCGCTGATGAC
	nicC R	GCTGGATGTCGGGATGGAAA
nicC flanking primer for mutant confirmation	nicC full F	GGTACCATGAAGCAACCCCGTATCGCGATCGTG
	nicC full R	AAG CTT GCC GGC CAC CGG CTC GCG
nicX partial fragment cloning in pK18mob	nicX F	GAATTC CTGAAGTCCGGCACCAAGAT
	nicX R	AAGCTT CCCCAGCGAGAACAGGAAAT
nicX flanking primer for mutant confirmation	nicX flk F	GATTCGCTGGCCTATCTCGG
	nicX flk R	TGTCGATATGGCAGGTGGTG
nicR partial fragment cloning in pK18mob	marR_F	GAATTC AAGGAAACCAGGGGCTGTTC
	marR_R	GGATCC GTCGCTCTCCAGCATCTTGT
nicR flanking primer for mutant confirmation	marR_flk F	GATCTTCCAGGAGGCGATCC
	marR_flk R	TCATTCGCCGTCGCTCTCCA
M13 primer	M13 fwd	TGTAAAACGACGGCCAGT
	M13 rev	AGGAAACAGCTATGACCAT
**qRT-PCR analysis**		
nicC	nicC_RT F	GAAGGTGCAGCTGGTCTC
	nicC_RT R	GCACGCCGAACACATCA
nicX	nicX_RT F	ACTATGTTTCCGAGCCGATC
	nicX_RT R	GAGATCGCATAACCTTCGGG
nicR	nicR_RT F	TCCCGCGATTCCTACGATTT
	nicR_RT R	GGTTTCCTTGACCGTGCAGA
flhC	flhC_RT F	AAATCACCCTGGCGATCGAA
	flhC_RT R	AGACGCCCTTGAGTTCCTTG
flhD	flhD_RT F	TCGCCGAGATCAAGGAAGTG
	flhD_RT R	CCGAGATTCCCATCCGGAAC
16S rRNA gene	16s fwd	GGAGTACGGTCGCAAGATTAAA
	16s rev	GACCATGTCAAGGGTAGGTAAG

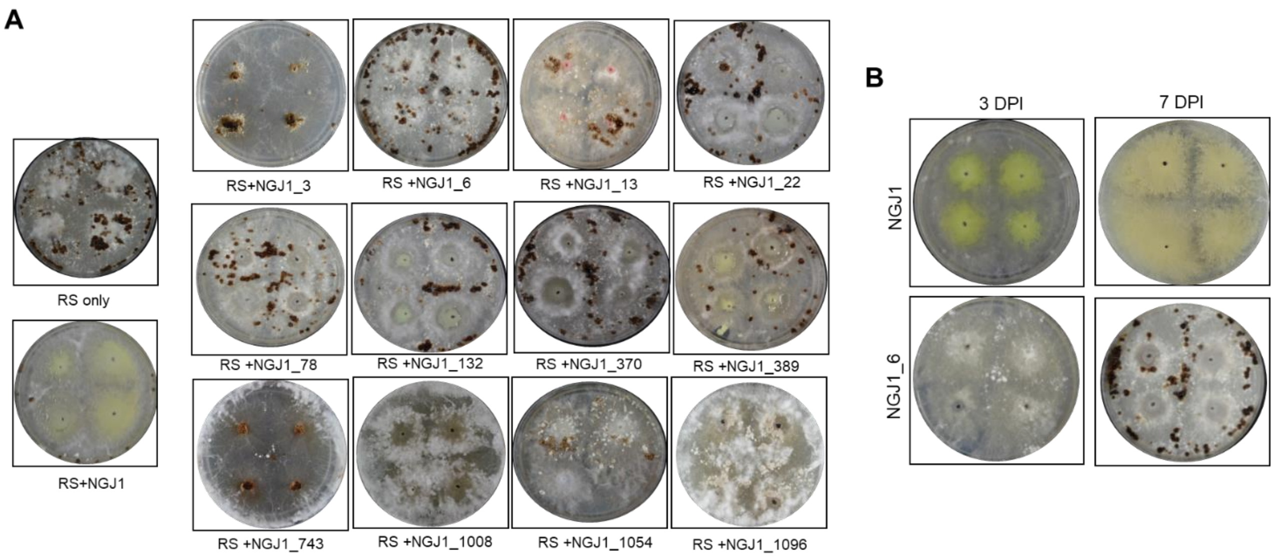

Figure S1: Transposon-tagged mutants of NGJ1 that are defective in mycophagy. Representative image of (A) interaction of transposon-tagged mutants of NGJ1 (10^3 cells/ml density) with *R. solani* (RS), at 7 DPI. (B) NGJ1_6 is defective in mycophagy against *R. solani* at both 3 and 7 DPI. The experiments were independently repeated three times with a minimum of three technical replicates.

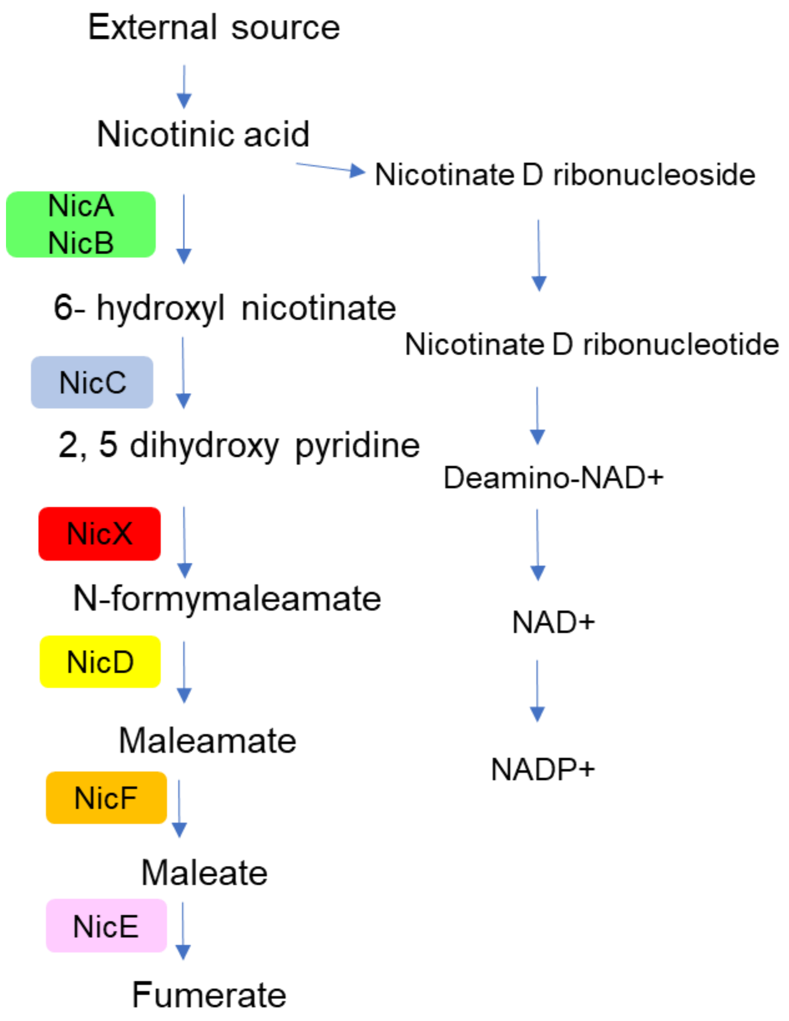

Figure S2: Genes associated with NA catabolic (nic) pathway in *B. gladioli*

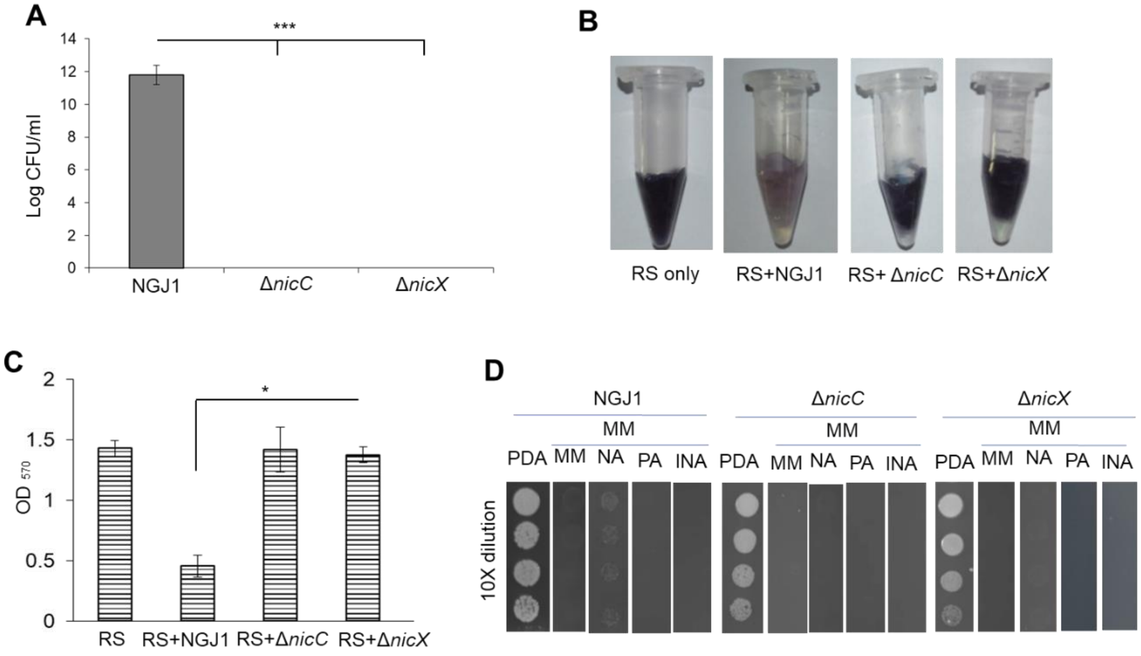

Figure S3: Characterization of Δ*nicC* and Δ*nicX* mutants of NGJ1. (A) Bacterial abundance on fungal mycelia, estimated by serial dilution plating and colony counting. (B) Representative image of MTT stained *R. solani* (RS) mycelia treated with different NGJ1 strains. (C) The spectrophotometric quantification of formazon (OD_{570}), accumulated in the MTT-stained fungal mycelia, with and without bacterial treatment. (D) Growth of NGJ1 strains in M9 minimal media (MM) with nicotinic acid (NA, 20 µM) as the sole carbon source. Aromatic compounds picolinic acid (PA, 20 µM) and isonicotinic acid (INA, 20 µM) were used as control. The experiments were independently repeated three times with a minimum of three technical replicates. Graphs show mean values ± standard error. Asterisks * and *** indicate a significant difference at $P < 0.05$ and $P < 0.001$ (estimated using one-way ANOVA), respectively.

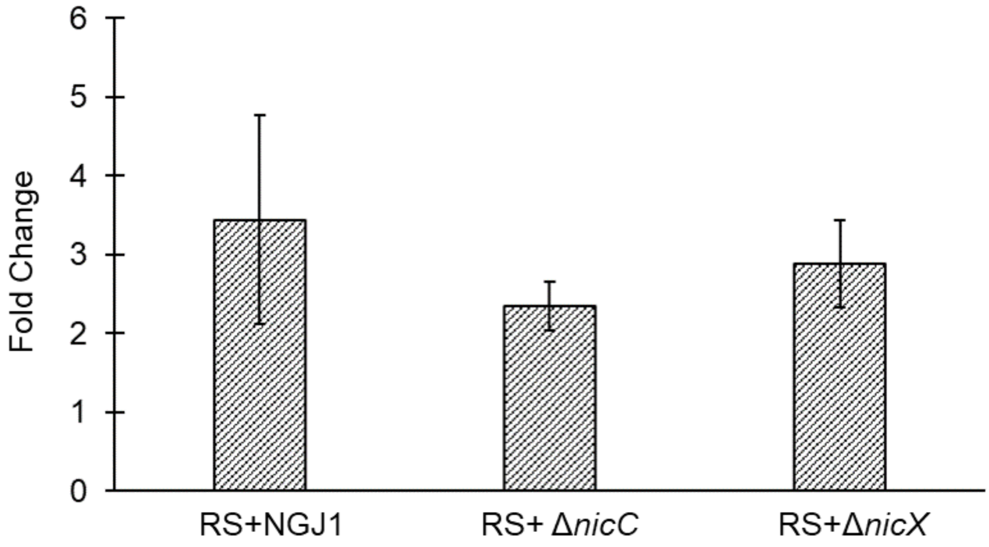

Figure S4: The qRT-PCR-based expression of *nicT* gene during 48 h of confrontation of NGJ1 strains with *R. solani*. The fold change ($2^{-\Delta\Delta Ct}$) in gene expression was calculated during 48 h confrontation of NGJ1 strains with *R. solani* (RS) to that of NGJ1 strains grown alone (without fungi), using 16S rRNA of NGJ1 as endogenous control. The experiments were independently repeated three times with three technical replicates. Graphs show mean values ± standard error of three biological replicates.

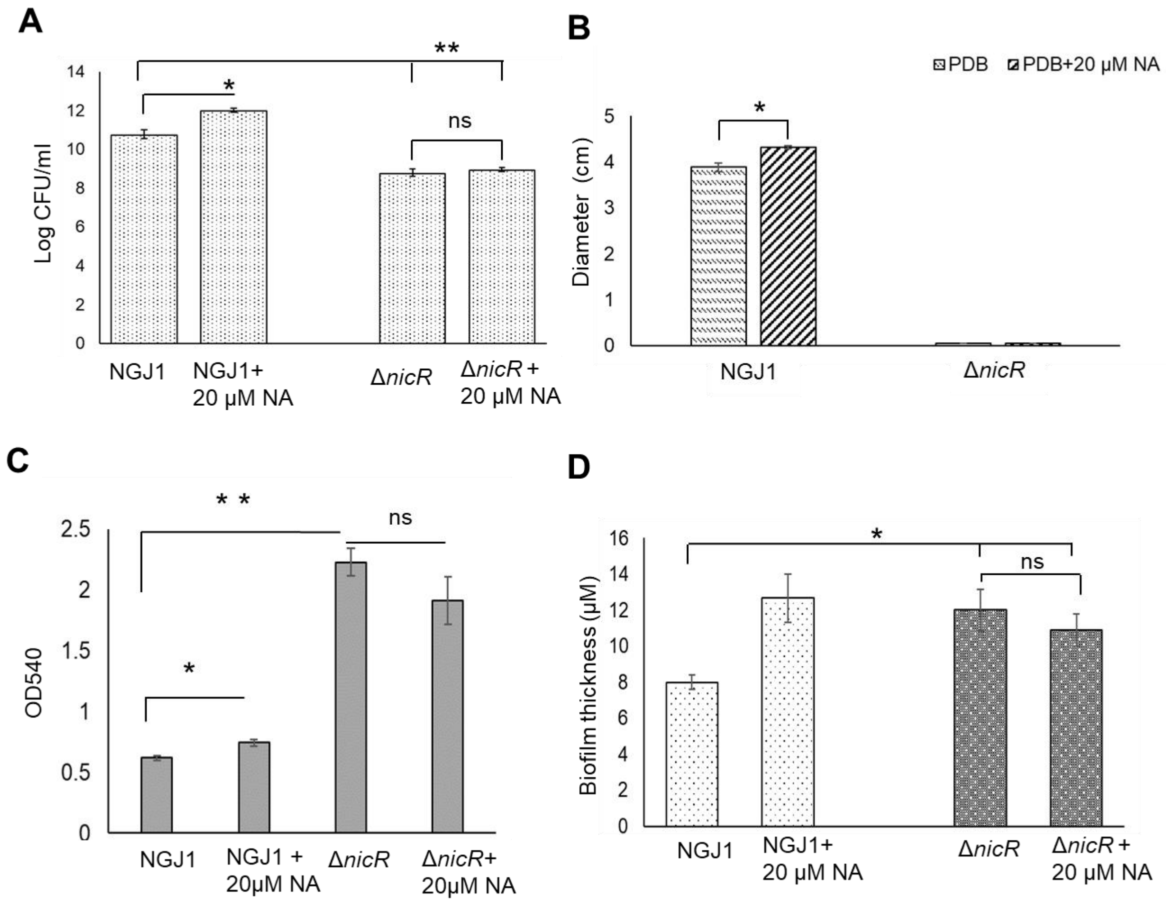

Figure S5: Characterization of Δ*nicR* mutant of NGJ1 (A) Bacterial abundance, represented as colony forming count (CFU), in the bacterial-fungal interaction zone. (B) The diameter of the motility zone on the semi-solid swim plates after 16 h of growth. (C) Spectrophotometric (OD_{540}) quantification of crystal violet stain extracted from the surface attached bacterial cells grown for 72 h under static conditions. (D) Average biofilm thickness formed by NGJ1 and Δ*nicR* strains estimated using confocal laser-scanning microscopic (CLSM) images (n-15). Graphs show mean values ± standard error of three independent replicates. Asterisks * and ** indicate a significant difference at $P < 0.05$ and $P < 0.01$ (estimated using one-way ANOVA). ns= non-significant.
